# Synergistic Polyphenol Recovery From *Embelia ribes* Using MP‐UAE, UAE, and MAE and In Silico Studies Against HCV Protease

**DOI:** 10.1155/jamc/5570481

**Published:** 2026-09-28

**Authors:** Faiza Imtiaz, Dildar Ahmed, Saira Zumar, Samiah H. Al-Mijalli, Munawar Iqbal

**Affiliations:** ^1^ Department of Chemistry, Forman Christian College; A Chartered University, Lahore, Pakistan; ^2^ Department of Chemistry, The University of Lahore, Lahore, Pakistan, uol.edu.pk; ^3^ Department of Biology, College of Sciences, Princess Nourah bint Abdulrahman University, P. O. Box 84428, Riyadh 11671, Saudi Arabia, pnu.edu.sa; ^4^ Zero Emission Technologies Innovation Center, University of Tabuk, Tabuk 47913, Saudi Arabia, ut.edu.sa; ^5^ Department of Chemistry, Faculty of Science, University of Tabuk, Tabuk 47913, Saudi Arabia, ut.edu.sa

**Keywords:** *Embelia ribes*, modern bioactive compound extraction, response surface methodology

## Abstract

*Embelia ribes* (ER) is well regarded in herbal medicine for its therapeutic properties, making the development of efficient methods to obtain its bioactive extracts of high yield essential. This study evaluated and optimized three extraction techniques, microwave‐pretreated ultrasound‐assisted extraction (MP‐UAE), ultrasound‐assisted extraction (UAE), and microwave‐assisted extraction (MAE), to isolate bioactive compounds from ER seeds. Embelin, a primary bioactive, was quantified in the optimized extracts, while 13 bioactives, including embelin, were analyzed through in silico studies targeting HCV NS3 protease. Process optimization using response surface methodology (RSM) focused on key parameters: total phenolic content (TPC), total flavonoid content (TFC), DPPH radical scavenging activity (DRSA), and ABTS radical scavenging activity (ARSA). Experimental results validated the models, with MP‐UAE yielding the highest TPC (183.00 mg GAE/g DW), TFC (54.54 mg RE/g DW), DRSA (72.80%), and ARSA (81.33%). Optimized conditions included microwave power of 238 W, 48‐min sonication, and a 19‐mL/g solvent‐to‐plant ratio for MP‐UAE. Embelin content, measured spectrophotometrically, was highest in MP‐UAE at 39.98 ± 0.97 mg/g DW. Molecular docking using Maestro 12.5 identified amentoflavone (−8.991 kcal/mol), embelin (−8.152 kcal/mol), quercetin (−8.107 kcal/mol), and aloe‐emodin (−8.014 kcal/mol) as top compounds, surpassing the standard drug boceprevir (−5.342 kcal/mol). This study establishes a green, efficient extraction method for ER seed bioactives, highlighting the antiviral potential of embelin and contributing to herb‐based pharmaceutical advancements, particularly in antiviral drug development.

## 1. Introduction

*Embelia ribes* (ER) is a medicinal plant that has been highly valued for its wide‐ranging healing properties, including antimicrobial, anti‐inflammatory, antioxidant, and anthelmintic effects [1–3]. Rich in bioactive compounds like embelin, aloe‐emodin, amentoflavone, vilangin, quercetin, and β‐sitosterol, it has been used to address various health issues, including digestive disorders, respiratory ailments, skin infections, and parasitic diseases [4, 5]. Recent scientific studies have further supported its medicinal value, revealing its anticancer and neuroprotective potential, thus confirming its long‐standing role in holistic healing practices [6]. Known for its powerful detoxifying properties, ER plays a crucial role in enhancing immune function and promoting overall health. Additionally, its effectiveness in managing metabolic disorders, such as diabetes, underscores its significance in modern medicine [7]. Notably, embelin is known for its potential to modulate various cellular pathways [7]. Therefore, optimizing extraction methods for these compounds is crucial to maximize their pharmacological efficacy and therapeutic potential.

Microwave‐assisted extraction (MAE) and ultrasonic‐assisted extraction (UAE) are modern, nonconventional extraction techniques that provide numerous advantages over traditional methods [8]. MAE utilizes microwave energy to heat the solvent and plant matrix, leading to rapid cell wall disruption and efficient release of intracellular compounds [9]. UAE, on the other hand, employs ultrasonic waves to generate cavitation bubbles within the solvent, causing physical agitation that enhances solvent penetration and improves mass transfer [10]. The integration of these techniques in the form of microwave‐pretreated ultrasound‐assisted extraction (MP‐UAE) combines the strengths of both, resulting in superior extraction yields and reduced extraction times [11]. This synergy occurs because the microwaves enhance thermal effects, while ultrasound‐induced cavitation further disrupts plant cell structures, making it an ideal method for extracting sensitive phytochemicals [12].

Response surface methodology (RSM) is utilized to systematically evaluate and optimize the various parameters influencing extraction. RSM is a robust statistical tool that aids in designing experiments, constructing models, and assessing the effects of multiple variables simultaneously [13]. RSM enables the identification of optimal conditions by examining interactions between variables like microwave power, ultrasound time, and solvent concentration. This approach enhances extraction efficiency and offers valuable insights into the underlying mechanisms of the process [14]. Various studies have demonstrated that modern extraction techniques, such as MAE and UAE, are effective for extracting bioactive compounds from ER [15–17]. However, the potential benefits of combining these advanced methods with RSM optimization have yet to be explored. Such an approach could significantly enhance the extraction yield, offering a promising avenue for improving the efficiency of bioactive compound recovery from ER.

The NS3/4A protease is a critical enzyme in the replication of the hepatitis C virus (HCV) and is targeted by antiviral drugs to inhibit viral replication [18]. Molecular docking simulations allow for the prediction of the binding interactions between ligands and target proteins, providing insights into the potential inhibitory activity of the compounds against viral enzymes [19–21]. By analyzing the docking scores and binding conformations, the researchers aim to identify compounds that can act as effective HCV protease inhibitors. This integrated approach not only validates the biological activity of the extracted phytochemicals but also highlights their potential as lead compounds for drug development [20, 22, 23].

This study presents a novel and integrative strategy by employing MP‐UAE for the first time to efficiently isolate embelin and other key phytochemicals from ER seeds. Using RSM, extraction parameters were systematically optimized to maximize the yield and recovery of bioactive compounds. A key distinguishing feature of this research is the novel integration of extraction optimization with molecular docking analyses to evaluate the antiviral potential of the isolated phytochemicals against the HCV NS3 protease. This dual‐focused approach not only advances green extraction technologies but also provides meaningful contributions to the discovery of potential antiviral agents, marking a significant step forward in both phytochemical and therapeutic research.

## 2. Materials and Methods

### 2.1. Sample Collection and Preparation for Extracts

Seeds of ER were collected from a Lahore market in Pakistan and identified by Dr Zaib Un Nisa, Assistant Professor, Department of IMBB/CRiMM, University of Lahore, Pakistan. The samples were washed with distilled water and dried at room temperature in the shade for 15 days. After that, the plant material was ground to a fine powder using a Clatronic grinder. The resulting powder (ERP) was stored in a refrigerator until further use.

### 2.2. Experimental Designs for Extraction

The selection of technological input variables for the optimization process, such as microwave power, sonication temperature and time, and solvent‐to‐plant powder ratio (SPR), was based on a combination of prior literature, preliminary trials (single‐factor experiments), and equipment capabilities. For each extraction technique (UAE, MAE, and MP‐UAE), three variables were selected and evaluated at three levels (−1, 0, +1), as outlined in Table 1.

**TABLE 1:** MP‐UAE, MAE, and UAE experimental designs.

Extraction techniques	Input variables	Units	Levels		
			−1	0	1
UAE	Sonication temperature	(°C)	30	40	50
	Time	(min)	30	45	60
	Solvent‐to‐plant powder ratio	(mL/g)	15	25	35

MAE	Microwave power	(W)	160	320	480
	Time	(s)	30	60	90
	Solvent‐to‐plant powder ratio	(mL/g)	15	25	35

MP‐UAE	Microwave power	(W)	160	320	480
	Sonication time	(min)	30	45	60
	Solvent‐to‐plant powder ratio	(mL/g)	15	25	35

Based on the above selection, a three‐factor, three‐level Box–Behnken design (BBD) of RSM was applied to optimize the extraction process and to analyze the effects of different variables on outcomes such as total phenolic content (TPC), total flavonoid content (TFC), DPPH radical scavenging activity (DRSA), and ABTS radical scavenging activity (ARSA). For MP‐UAE, the input variables included microwave power, sonication time, and the SPR. For MAE, the input variables included power, irradiation time, and the SPR, while for UAE, the key variables were temperature, time, and SPR.

### 2.3. MP‐UAE

MP‐UAE was conducted using a microwave oven (Panasonic NN‐SF564WQPQ 27LTR), with parameters set at power levels of 160, 320, and 480 W, while maintaining a constant irradiation time of 120 s with varying SPR as reported in Table 1. Each extraction run underwent sonication in an ultrasonic bath for 30, 45, and 60 min, respectively, to optimize extraction efficiency by enhancing the interaction between the solvent and the plant material. This combination of microwave and sonication energy aimed to improve the overall yield and quality of the extracted compounds. Following extraction, the extracts from all 17 runs were filtered using a funnel to separate the liquid phase. The filtrates were subsequently dried in an oven at a controlled temperature and then transferred to preweighed centrifuge tubes for accurate sample handling. The dried extracts were reconstituted to a concentration of 2.5 mg/mL using an appropriate solvent, ensuring uniformity across all samples for subsequent analyses.

### 2.4. MAE

For MAE, a specified amount of dried plant material (ERP) was placed in a conical flask, and a measured volume of 70% hydroethanol was added. After thoroughly mixing, the flask was positioned in a microwave oven for treatment according to the protocol outlined in Table 1. Following microwave exposure, the contents of the flask were filtered to obtain the extract as a clear filtrate. The solvent in the filtrate was then evaporated using a rotary evaporator (SCILOGEX SCI100‐S). The resulting extract was dried in an oven (Memmert UNB 100, Lahore, Pakistan) and stored in a refrigerator for future use.

### 2.5. UAE

UAE was conducted using a Hielscher ultrasound processor (200 W, 26 kHz), following a previously established method. The flask containing the plant powder soaked in 70% hydroethanol was subjected to ultrasound treatment for varying durations as specified in the experimental design, maintaining a controlled temperature of 40 ± 2°C. The subsequent steps remained the same as those outlined for MAE.

### 2.6. Assays for TPC and TFC

The methodologies used to determine TPC and TFC were based on previously established procedures [24]. TPC is expressed as milligrams of gallic acid equivalents per gram of dry weight (mg GAE/g DW), while TFC is reported as milligrams of rutin equivalents per gram of dry weight (mg RE/g DW). The results are presented in Tables 2 (for MAE) and Tables 3 (for UAE).

**TABLE 2:** MP‐UAE experimental design and observed results.

Runs	Microwave power (W)	SPR (mL/g)	Sonication time (min)	TPC (mg GAE/g DW)	TFC (mg RE/g DW)	DPPH (%)	ABTS (%)
1	320	35	30	93.73	43.55	67.77	69.66
2	480	15	45	76.51	29.77	80.94	76.22
3	480	35	45	69.51	26.66	31.68	61.66
4	320	25	45	187.06	45.33	49.65	88.61
5	320	25	45	180.17	41.33	58.31	86.66
6	160	25	60	138.84	32.11	57.44	89.50
7	480	25	60	75.28	27.33	70.85	78.41
8	320	35	60	128.95	54.77	66.98	88.88
9	160	25	30	114.95	28.22	70.43	83.55
10	320	25	45	182.62	44.33	54.27	83.33
11	160	15	45	157.95	25.55	64.27	87.66
12	320	15	60	151.62	40.88	96.95	91.90
13	320	25	45	181.40	44.01	56.35	87.27
14	160	35	45	139.95	53.77	71.63	90.27
15	480	25	30	32.84	18.88	40.12	61.11
16	320	25	45	181.06	38.66	62.26	84.44
17	320	15	30	99.06	32.00	78.07	87.50

### 2.7. Antioxidant Assays

Antioxidant or free radical scavenging activities were evaluated using the DPPH [25] and ABTS [26] assays following methods that have been previously reported without any modifications.

### 2.8. Estimation of Total Embelin Content

The concentration of embelin in optimized extracts of each studied technique was calculated using a reported method [27]. A standard solution of embelin was prepared by dissolving 10 mg of the compound in a 100‐mL volumetric flask containing methanol, achieving a final concentration of 100 ppm. A series of dilutions (10–90 ppm) was made by withdrawing 1–9 mL of the standard solution and diluting it in 10‐mL volumetric flasks with methanol. Absorbance was measured at 288 nm using a UV‐visible spectrophotometer [28]. A calibration plot was created, and a linear regression equation was obtained (*y* = 0.0105*x* + 0.0937; *R* ^2^ = 0.999) that was used for embelin quantification in the extracts.

### 2.9. Molecular Docking Studies

#### 2.9.1. Ligand and Enzyme Preparation

Docking studies were performed for 13 compounds reported from ER and the standard drug molecule, Boceprevir (Table 3, Figure 1) [4, 29, 30]. The crystal structure of the NS3/4 protease receptor, complexed with the macrocyclic F9K inhibitor (PDB ID: 4A92) at a resolution of 2.73 Å, was retrieved from the Protein Data Bank. The receptor molecule was prepared for docking studies using the “Protein Preparation Wizard” in the Maestro interface, followed by a refinement step. Water molecules in the receptor were removed, hydrogens were added, and energy minimization was performed using the OPLS3 force field. A grid box was generated by centering it on the co‐crystallized ligand F9K. Ligand preparation was carried out with the LigPrep module in Maestro, utilizing the OPLS force field to achieve low‐energy conformations [31].

**TABLE 3:** Predicted and observed results.

Sr. No.		Microwave power (W)	SPR (mL/g)	Sonication time (min)	Temperature (°C)	Microwave irradiation time (s)	TPC (mg GAE/g DW)	TFC (mg RE/g DW)	DPPH (%)	ABTS (%)
MP‐UAE	Predicted	238	19	48	—		187.437	37.38	64.42	90.45
	Observed						**182.998**	**44.54**	**69.81**	**86.33**

MAE	Predicted	311	30		—	51	179.64	45.86	62.69	60.34
	Observed						175.46	42.21	61.89	61.17

UAE	Predicted	—	19	60	36		124.064	45.362	61.417	55.701
	Observed						125.36	52.098	59.354	59.58

*Note:* The bold values represent the optimized responses obtained using the best‐performing technique among the three techniques evaluated.

**FIGURE 1:**
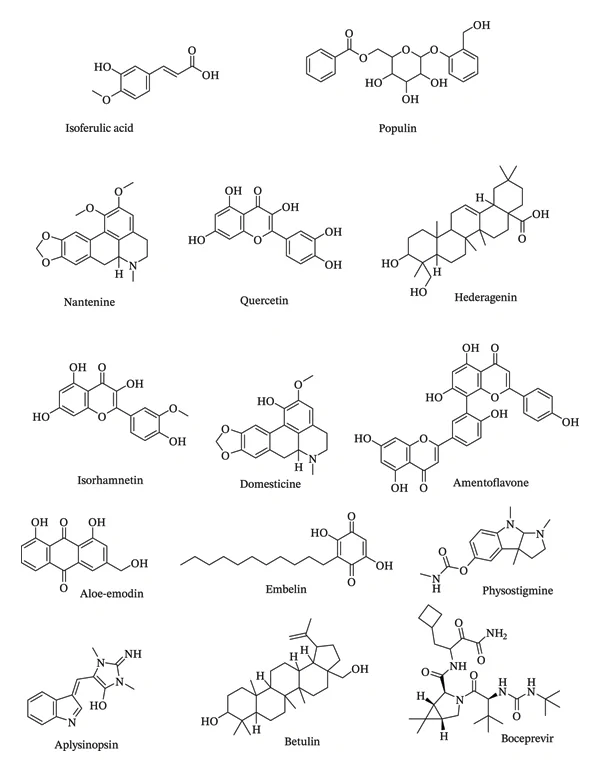
Chemical structures of the compounds used for molecular docking.

#### 2.9.2. Molecular Docking Studies

Molecular docking was conducted using the Grid‐based Ligand Docking with Energetics (GLIDE) tool (Schrödinger Inc., LLC, New York, NY, 2008) to analyze ligand interactions within the binding pocket of the NS3/4A protease kinase receptor. Docking was performed using the extra precision (XP) algorithm, allowing the ligands to be ranked according to their specific conformations within the receptor. Finally, the best poses were selected based on their computed glide scores and ranked according to their E model values. After docking, the results were analyzed by Discovery Studio 2024.

#### 2.9.3. Investigation of Drug‐Likeness Score and Physicochemical Properties

In this study, we selected phytochemicals present in ER to evaluate their drug‐likeness according to Lipinski’s Rule of Five (Ro5). Lipinski’s rule was followed to assess the drug‐like properties of each selected compound [32]. The data on drug‐likeness were compiled in accordance with Lipinski’s criteria. Canonical Simplified Molecular Input Line Entry System (SMILES) was obtained from PubChem and analyzed using the Molsoft software, available online at https://www.molsoft.com/mprop/, to gather data and calculate descriptors and the drug‐likeness scores are presented in Table 4 [33]. The SMILES representations of the compounds with the lowest binding energies were submitted to the SwissADME online server for an analysis of their pharmacokinetics and drug‐likeness properties. Molecules were filtered based on two key molecular properties: a water partition coefficient (WlogP) below 5, which predicts low toxicity, reduced nonspecific binding, and suitability for potential oral administration, and a topological polar surface area (TPSA) under 140 Å^2^, indicating a high likelihood of complete absorption [34]. Based on these criteria, ligands that demonstrated strong interactions with the target protein were selected. These ligands were then further analyzed for bioavailability using the BOILED‐Egg model. For toxicity evaluation of each molecule, the SMILES for each compound was then fed into the StopTox software that was freely available on https://stoptox.mml.unc.edu/ (Table 5) [35].

**TABLE 4:** Compounds with their molecular docking results.

Compound name	Symbol	Molecular formula	Docking score (kcal/mol)	Glide energy (kcal/mol)	Interacting amino acids	Drug‐likeness score model score (Lipinski’s RO5)/BBB score
Isoferulic acid	ER‐1	C_10_H_10_O_4_	−4.326	−14.4	SER^139^, LEU^135^, GLY^58^, HIS^57^	−0.93/2.97
Populin	ER‐2	C_20_H_22_O_8_	−6.14	−29.882	ASP^437^, VAL^436^, ARG^161^, GLY^162,^ ASP^487^, PHE^531^, PHE^438^, MET^623^	−0.63/2.41
Nantenine	ER‐3	C_20_H_21_NO_4_	−4.46	−30.474	ASP^487^, CYS^525^, VAL^524^, GLN^526^, ASP^81^, PHE^486^, ARG^155^, HIS^57^, ALA^156^	0.42/5.27
Quercetin	ER‐4	C_15_H_10_O_7_	**−8.107**	−17.841	GLN^41^, HIS^57^, SER^139^, SER^138^, LEU^135^, ALA^156^	0.52/2.55
Hederagenin	ER‐5	C_30_H_48_O_4_	−3.688	−25.021	ASP^81^, HIS^57^, ARG^481^	0.47/2.95
Isorhamnetin	ER‐6	C_16_H_12_O_7_	−6.914	−26.443	GLY^137^, SER^138^, SER^139^, ALA^156^, HIS^57^	0.39/2.66
Domesticine	ER‐7	C_19_H_19_NO_4_	−4.622	−26.183	CYS^525^, PHE^486^, GLN^526^, VAL^524^, HIS^57^, ASP^81^, MET^485^	0.60/4.92
Amentoflavone	ER‐8	C_30_H_18_O_10_	**−8.991**	−43.759	PHE^486^, GLN^41^, GLY^137^, SER^138^, SER^139^, LEU^135^, ALA^157^, ASP^81^, HIS^57^, MET^485^	0.19/0.51
Aloe‐emodin	ER‐9	C_15_H_10_O_5_	**−8.014**	−27.727	SER^138^, SER^139^, GLN^41^, ALA^157^, LYS^136^, HIS^57^	0.01/2.93
Embelin	ER‐10	C_17_H_26_O_4_	**−8.152**	−28.343	GLY^137^, SER^138^, SER^139^, ALA^157^, ALA^156^, HIS^57^, VAL^524^	−0.05/2.52
Physostigmine	ER‐11	C_15_H_21_N_3_O_2_	−1.079	−25.614	MET^485^, ASP^487^, ALA^156^, VAL^158^, ARG^155^, HIS^57^, VAL^524^	1.07/5.03
Aplysinopsin	ER‐12	C_14_H_14_N_4_O	−6.029	−25.483	ASP^79^, ASP^168^, ARG^155^, VAL^524^, MET^485^, CYS^525^	−1.69/4.82
Betulin	ER‐13	C_30_H_50_O_2_	−4.19	−29.559	CYS^159^, PHE^486^, ALA^157^, ALA^156^, VAL^132^, VAL^524^, MET^485^,	−0.13/4.03
Boceprevir	BP	C_27_H_45_N_5_O_5_	−5.342	−38.833	GLY^137^, HIS^57^, GLN^526^, ALA^156^, ARG^155^, PHE^154^, LYS^136^	0.21/0.78

*Note:* The bold values represent the highest docking scores among the studied molecules.

**TABLE 5:** StopTox toxicity parameters of the selected compounds.

Symbol	Acute inhalation toxicity	Acute oral toxicity	Acute dermal toxicity	Eye irritation and corrosion	Skin sensitization	Skin irritation and corrosion
ER‐1	Nontoxic	Nontoxic	Nontoxic	Toxic	Sensitizer	Negative
ER‐2	Nontoxic	Nontoxic	Nontoxic	Toxic	Sensitizer	Negative
ER‐3	Nontoxic	Toxic	Nontoxic	Nontoxic	Nonsensitizer	Negative
ER‐4	Nontoxic	Nontoxic	Toxic	Toxic	Sensitizer	Negative
ER‐5	Nontoxic	Nontoxic	Nontoxic	Nontoxic	Nonsensitizer	Negative
ER‐6	Nontoxic	Nontoxic	Toxic	Nontoxic	Sensitizer	Negative
ER‐7	Nontoxic	Toxic	Nontoxic	Nontoxic	Sensitizer	Negative
ER‐8	Nontoxic	Nontoxic	Toxic	Toxic	Sensitizer	Negative
ER‐9	Nontoxic	Nontoxic	Nontoxic	Toxic	Sensitizer	Negative
ER‐10	Nontoxic	Nontoxic	Nontoxic	Nontoxic	Sensitizer	Negative
ER‐11	Nontoxic	Toxic	Toxic	Toxic	Nonsensitizer	Negative
ER‐12	Nontoxic	Toxic	Nontoxic	Toxic	Sensitizer	Negative
ER‐13	Nontoxic	Toxic	Nontoxic	Nontoxic	Nonsensitizer	Negative
BP	Nontoxic	Nontoxic	Nontoxic	Toxic	Nonsensitizer	Negative

## 3. Results and Discussion

### 3.1. MP‐UAE

Table 2 shows the experimental design for MP‐UAE and its results. The effect of the individual input variables is discussed below.

#### 3.1.1. MP‐UAE Effects on Responses

##### 3.1.1.1. Effects on TPC and TFC

The highest TPC of 187.06 mg GAE/g DW was obtained under moderate microwave power (320 W), SPR of 25 mL/g, and a sonication time of 45 min. The MP‐UAE technique demonstrated significant variations in the extraction yields of TPC and TFC depending on the balance between microwave power, sonication time, and SPR. This outcome aligns with previous studies, which report that moderate microwave power allows effective solvent penetration and heat distribution, leading to higher phenolic release without causing thermal degradation [36]. Increasing the power to 480 W, however, resulted in a dramatic drop in TPC to 32.84 mg GAE/g DW at 25 SPR and 30‐min sonication time. Similar findings were reported, as high microwave power can generate excessive heat, breaking down cell walls too rapidly and leading to the degradation of phenolic compounds [37, 38].

Figures 2 and 3 illustrate the 3D response surface interactions between the input variables and their corresponding responses for MP‐UAE. The SPR ratio plays a crucial role in determining the extraction efficiency of this combined technique. At lower SPR values, the TPC is typically low due to insufficient solvent to effectively extract phenolic compounds from the plant matrix. As the SPR increases, TPC yields also rise, reaching a maximum at 25 mL/g. However, beyond this point, at an SPR of 35 mL/g, TPC starts to decline. This decrease may occur because excessive dilution of the phenolic compounds in the solvent can hinder their extraction efficiency, reducing the concentration of active compounds available for analysis [39]. Additionally, overly high solvent volumes can lead to less effective solvation and interaction between the solvent and phenolic analytes [39].

**FIGURE 2:**
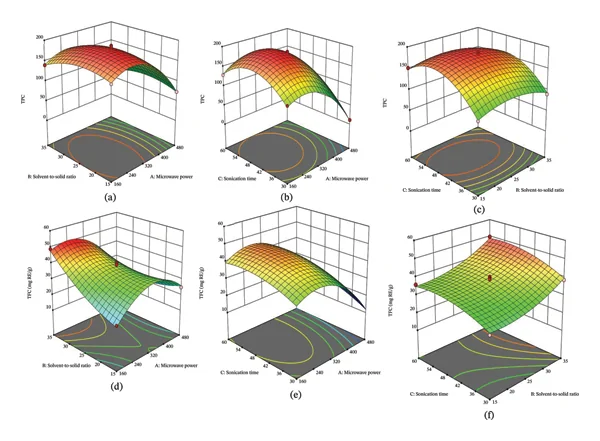
Microwave‐pretreated ultrasound‐assisted extraction: 3D response surface plots of effects of different variables on (i) total phenolic content (a–c); (ii) total flavonoid content (d–f).

**FIGURE 3:**
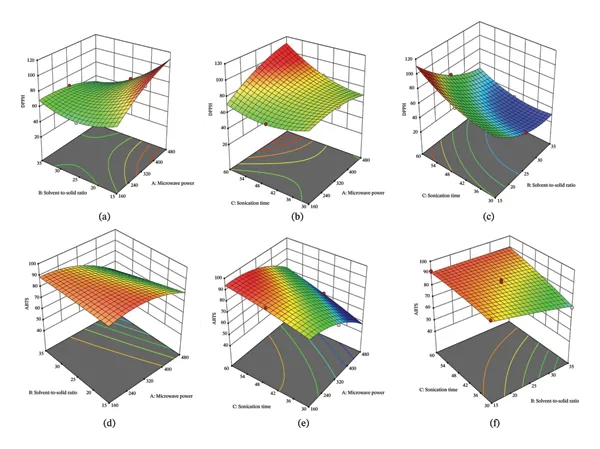
Microwave‐pretreated ultrasound‐assisted extraction: 3D response surface plots of effects of different variables on (i) DPPH‐RSA (a–c); (ii) ABTS‐RSA (d–f).

The variations in responses across different runs demonstrate that using both techniques together enhances the extraction of bioactive compounds, and their antioxidant potential compared to the use of each technique alone, due to improved solvent penetration and cell disruption. This synergy likely results in increased mass transfer and improved yield, as both microwave and ultrasound techniques complement each other in breaking down plant matrices and enhancing polyphenolic recovery [11]. When microwave pretreatment is followed by sonication, the initial heating and cell wall disruption from microwaves can prepare the sample for improved extraction. However, if conditions are too intense, the combined thermal and mechanical stress may lead to accelerated degradation, resulting in a reduced yield of bioactive compounds. Similar trends have been previously reported, where MP‐UAE treatment initially increases TPC yield, followed by a decrease after reaching a maximum [11, 40].

The TFC results followed a comparable trend, with the maximum yield of 54.77 mg RE/g DW achieved at 320 W and a prolonged sonication time of 60 min. The efficiency of MP‐UAE in flavonoid extraction at moderate power can be attributed to the synergistic effect of microwave and ultrasonic waves, which enhances cell disruption and accelerates solvent diffusion into the matrix [41]. However, high power (480 W) combined with extended sonication (60 min) showed a significant decline in TFC to 27.33 mg RE/g DW. This decrease may be due to structural changes and degradation of thermosensitive flavonoids under prolonged exposure to high‐energy conditions, consistent with already reported findings of flavonoid losses during intense microwave treatment [42].

##### 3.1.1.2. Effects on Antioxidant Activities

The antioxidant or radical scavenging activities, measured using DPPH and ABTS assays, showed a significant correlation with TPC and TFC values. The highest DRSA (96.95%) and ABTS scavenging (91.9%) were observed at moderate power (320 W) and prolonged sonication times, indicating efficient extraction of antioxidant compounds under optimal conditions. These results are supported by other reported studies, which concluded that moderate microwave and ultrasound power levels prevent the denaturation of antioxidant compounds, ensuring maximum radical scavenging capacity [43]. The lower activities observed at 480 W could be due to the partial breakdown of polyphenolic structures, reducing their antioxidant effectiveness.

### 3.2. MAE

Table 6 shows the experimental design for MAE and its results, and the effect of the individual input variables is discussed below.

**TABLE 6:** MAE experimental design and observed results.

Runs	SPR (mL/g)	Microwave power (W)	Time (min)	TPC (mg GAE/g DW)	TFC (mg RE/g DW)	DPPH (%)	ABTS (%)
1	25	320	60	173.22	44.22	62.16	59.99
2	15	320	90	66.77	36.40	63.03	60.11
3	35	320	90	188.78	35.17	65.78	67.55
4	15	320	30	69.01	32.68	62.42	56.5
5	35	160	60	233.22	55.56	61.18	57.55
6	25	320	60	179.88	45.31	62.76	61.38
7	35	320	30	121.00	38.22	64.23	60.05
8	25	320	60	165.47	46.10	61.96	59.77
9	25	480	30	111.52	42.67	64.18	62.44
10	25	320	60	165.44	46.21	62.21	61.02
11	25	160	90	138.78	34.88	58.49	57.55
12	25	160	30	52.33	28.42	56.92	53.83
13	25	320	60	178.76	44.44	61.51	59.16
14	15	160	60	116.55	33.34	56.20	53.92
15	15	480	60	161.53	54.56	64.30	62.90
16	25	480	90	137.62	33.31	61.85	63.33
17	35	480	60	231.00	44.62	62.97	65.38

#### 3.2.1. MAE Effects on Responses

##### 3.2.1.1. Effects on TPC and TFC

Figures 4 and 5 represent the 3D response surface plots of interactions between the input variables and their corresponding responses for MAE. The MAE method demonstrated that extraction efficiency is highly influenced by microwave power and extraction time. The highest TPC (233.22 mg GAE/g DW) was observed at a microwave power of 160 W with a moderate duration of 60 min.

**FIGURE 4:**
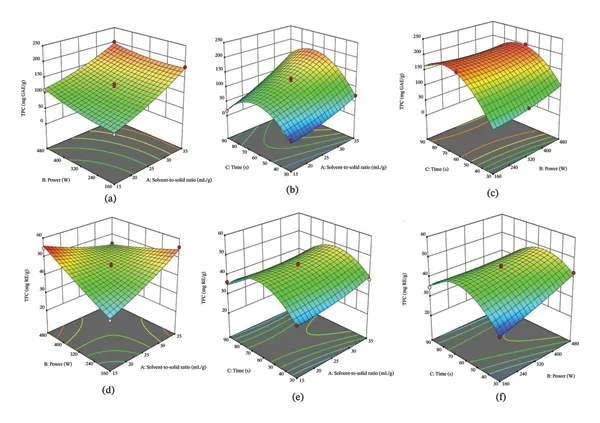
Microwave‐assisted extraction: 3D response surface plots of effects of different variables on (i) total phenolic content (a–c); (ii) total flavonoid content (d–f).

**FIGURE 5:**
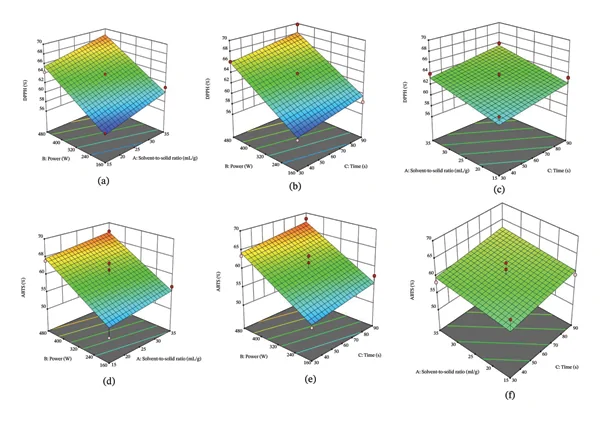
Microwave‐assisted extraction: 3D response surface plots of effects of different variables on (i) DPPH‐RSA (a–c); (ii) ABTS‐RSA (d–f).

This result is in accordance with the already reported studies that excessive power may cause the loss of volatile compounds and lead to the degradation of bioactive compounds [44]. This result suggests that, in this study, moderate power with sufficient duration provided optimal conditions for phenolic release, potentially due to minimized thermal degradation and effective cell wall disruption. The lowest TPC value (52.33 mg GAE/g DW) occurred at low microwave power (160 W) with a short extraction time of 30 min, underscoring the importance of both adequate power and duration for efficient extraction. This aligns with the reported literature, which highlights that insufficient power and time result in poor extraction due to limited cell wall breakdown and solvent penetration [45, 46]. The TFC values showed some variation across power levels, with the highest TFC (55.56 mg RE/g DW) obtained at 160 W for 60 min, indicating that moderate power may favor flavonoid stability [46]. This suggests that a controlled power level can effectively release flavonoids without causing thermal degradation, which may occur at higher power.

##### 3.2.1.2. Effects on Antioxidant Activities

The antioxidant activities, assessed through DPPH and ABTS assays, demonstrated a strong association with TPC, indicating phenolics’ primary contribution to antioxidant activities. The highest DPPH and ARSA values of 65.78% and 67.55 were obtained under moderate microwave power (320 W), SPR of 35 mL/g, and microwave irradiation time of 90 s. Moderate power is essential in the extraction process as it provides just the right amount of energy needed to disrupt cell walls and release phenolic compounds effectively. This precise control helps prevent excessive heat generation, which could potentially harm sensitive antioxidants [47]. By adjusting the microwave power, the extraction process can enhance the yield of bioactive compounds while conserving their antioxidant properties.

The SPR is another critical factor that impacts extraction efficiency. In this study, the highest DPPH and ABTS activities were obtained at high SPR, i.e., 35 mL/g D. A high SPR ensures that the solvent adequately interacts with the solid matrix, leading to improved extraction of phenolics and other beneficial metabolites [48]. This ratio is particularly important for achieving a more uniform distribution of the solvent, resulting in better extraction outcomes. It has been previously reported in numerous studies that TPC, TFC, and antioxidant activities are generally enhanced with an increase in the solid‐to‐solvent ratio [49, 50]. A higher ratio has been found to favor the extraction of these compounds, likely due to improved mass transfer and greater solvent availability.

An exposure time of 90s is also significant in this process as SPR. This timeframe effectively balances the need for adequate extraction while minimizing the risk of degrading sensitive compounds [51]. Within this period, the energy applied can mobilize phenolic compounds efficiently without causing thermal damage. Extending the exposure time could result in unwanted alterations to the chemical structure of these compounds, thereby adversely affecting their antioxidant activity.

### 3.3. UAE

Table 7 shows the experimental design for UAE and its results, and the effect of the individual input variables is discussed below.

**TABLE 7:** UAE experimental design and observed results.

Runs	SPR (mL/g)	Time (min)	Temperature (°C)	TPC (mg GAE/g DW)	TFC (mg RE/g DW)	DPPH (%)	ABTS (%)
1	25	45	40	80.28	51.75	61.57	67.05
2	35	30	40	153.50	46.41	62.48	51.88
3	35	45	50	138.81	53.42	78.62	65.27
4	25	60	30	220.51	41.14	51.89	57.22
5	15	45	50	110.70	57.38	54.94	73.61
6	25	60	50	174.12	54.59	65.18	56.88
7	15	45	30	54.42	46.55	58.72	61.11
8	25	30	50	179.51	36.57	47.86	49.08
9	25	30	30	97.51	38.48	54.47	44.88
10	25	45	40	72.44	46.55	52.10	68.11
11	35	45	30	183.51	48.79	69.24	73.38
12	25	45	40	69.51	48.33	58.91	68.61
13	25	45	40	78.87	45.54	62.50	69.61
14	35	60	40	216.11	63.35	73.69	76.66
15	15	30	40	87.93	57.78	51.39	69.22
16	15	60	40	118.63	58.22	57.63	54.16
17	25	45	40	87.28	46.11	60.95	66.50

### 3.4. UAE Effects on Responses

#### 3.4.1. Effects on TPC and TFC

The UAE method demonstrated that extraction efficiency is greatly influenced by temperature and sonication duration. The highest TPC (220.5 mg GAE/g DW) was achieved at a low temperature (30°C), an SPR of 25 mL/g, and a sonication time of 60 min, while the lowest TPC (54.4 mg GAE/g DW) was observed at temperature (30°C), an SPR of 15 mL/g, and a sonication time of 30 min.

Figure 6 displays 3D response surface plots depicting how TPC yield varies with changes in process variables. Overall, increasing sonication time tends to raise TPC yield, likely due to enhanced cell wall disruption, which facilitates the release of phenolic compounds. Moreover, extended sonication improves solvent penetration into the plant material, enabling more efficient extraction of TPC [52, 53].

**FIGURE 6:**
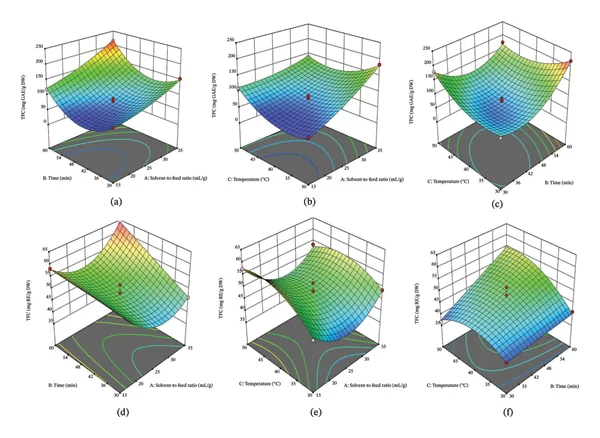
Ultrasound‐assisted extraction: 3D response surface plots of effects of different variables on (i) total phenolic content (a–c); (ii) total flavonoid content (d–f).

Increasing the SPR initially boosts TPC yield by enhancing the solubility and diffusion of phenolic compounds through ultrasonic cavitation, which disrupts cell walls and improves solvent penetration [54]. However, beyond an optimal ratio, this effect diminishes as dilution reduces extraction efficiency, and excess solvent can lead to oxidative degradation of sensitive phenolics, ultimately decreasing TPC yield [55, 56]. The highest TPC was observed at relatively low‐temperature conditions likely preventing the thermal degradation of phenolic compounds, which are sensitive to high temperatures [57]. This result is supported by various studies, which reported that UAE at low temperatures preserves phenolic integrity while maximizing cell wall disruption through cavitation effects [58]. This trend was also observed by earlier studies, which noted that flavonoids, particularly those with complex phytoconstituents, can degrade under prolonged ultrasound exposure at elevated temperatures [59, 60]. Thus, temperature control is critical in the UAE to optimize flavonoid recovery without compromising compound stability.

#### 3.4.2. Effects on Antioxidant Activities

The antioxidant activities (DPPH and ABTS) followed the trend of TPC and TFC, with the highest values (DPPH: 78.62% and ABTS: 76.66%) observed at 35 mL/g SPR at elevated time and temperature (Figure 7). This suggests that simple phenolics and flavonoids contribute to the antioxidant potential of the extracts [23]. As per reported literature, UAE promotes the release of low‐molecular‐weight antioxidant compounds that contribute significantly to radical scavenging activities [61]. The relatively lower activities at 50°C further support the notion of thermal degradation, as described by different studies that found that prolonged ultrasonic treatment at high temperatures can break down antioxidant molecules, reducing their efficacy [62, 63]. Therefore, UAE, when conducted at optimal temperature and sonication times, proves to be an effective technique for extracting thermally sensitive bioactive compounds from plant materials.

**FIGURE 7:**
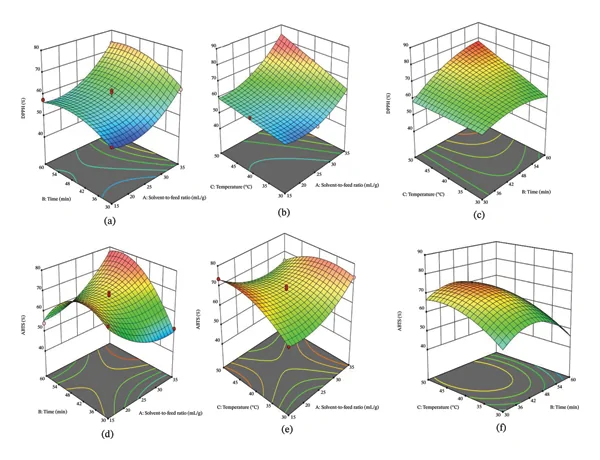
Ultrasound‐assisted extraction: 3D response surface plots of effects of different variables on (i) DPPH‐RSA (a–c); (ii) ABTS‐RSA (d–f).

### 3.5. Model Fitting and Optimization

#### 3.5.1. MP‐UAE Model Fitting and Optimization

MP‐UAE shows excellent model performance across all parameters, including TPC, TFC, DRSA, and ARSA (Table 8). Each parameter fits a quadratic model with very high *R* ^2^ values (e.g., TPC *R* ^2^ = 0.997, meaning the model explains 99.7% of TPC variability). The highly significant model *p* values (< 0.0001 for TPC, DRSA, and ARSA) indicate strong statistical reliability. Furthermore, the lack‐of‐fit *p* values (0.1677–0.9661) are relatively high, showing minimal discrepancy between the model’s predictions and actual data. These results suggest that MP‐UAE is highly effective for extracting bioactive compounds with low error, particularly for phenolics and flavonoids.

**TABLE 8:** Fit statistics for the obtained results.

**MP-UAE**				
**Terms**	**TPC**	**TFC**	**DRSA**	**ARSA**
	**Quadratic**	**Quadratic**	**Quadratic**	**Quadratic**

Lack‐of‐fit *p* value	0.1677	0.5644	0.9661	0.4425
Model *p* value	< 0.0001	0.0001	< 0.0001	< 0.0001
*R* ^2^	0.9975	0.9723	0.9754	0.9773
Adjusted *R* ^2^	0.9942	0.9368	0.9437	0.9480

**MAE**				
**Model**	**Quadratic**	**Quadratic**	**Quadratic**	**Linear**

Lack‐of‐fit *p* value	0.1688	0.1025	0.2540	0.1622
Model *p* value	< 0.0001	< 0.0001	< 0.0001	< 0.0001
*R* ^2^	0.9874	0.9854	0.9804	0.8835
Adjusted *R* ^2^	0.9711	0.9666	0.9552	0.8566

**UAE**				
**Model**	**Quadratic**	**Quadratic**	**Quadratic**	**Quadratic**

Lack‐of‐fit *p* value	0.4696	0.8221	0.9270	0.1579
Model *p* value	< 0.0001	0.0003	0.0028	< 0.0001
*R* ^2^	0.9925	0.9640	0.9300	0.9856
Adjusted *R* ^2^	0.9825	0.9178	0.8401	0.9670

#### 3.5.2. MAE Model Fitting and Optimization

MAE employs a mixed modeling approach. TPC and TFC are best represented by a quadratic model, while DRSA and ARSA follow a linear model (Table 8). The *R* ^2^ values for TPC (0.9874), TFC (0.9854), and DRSA (0.9804) are high, indicating strong predictability for phenolic and flavonoid contents. However, the *R* ^2^ values for ARSA (0.8835) are relatively lower, suggesting reduced predictive accuracy for antioxidant activities. Although all model *p* values are significant (< 0.0001), the adjusted *R* ^2^ values for ARSA (0.8238) reflect a weaker fit, implying that MAE may be less effective in maximizing antioxidant yields compared to MP‐UAE and UAE. Nevertheless, MAE remains an effective method, particularly for extracting phenolic and flavonoid compounds.

#### 3.5.3. UAE Model Fitting and Optimization

For UAE, a quadratic model was found to best fit all the responses (TPC, TFC, DRSA, and ARSA) with *R* ^2^ values above 0.93, showing strong correlations (Table 8). The adjusted *R* ^2^ values for TPC and TFC were 0.9825 and 0.9178, respectively, demonstrating the model’s reliability. For DRSA and ARSA, the adjusted *R* ^2^ values were slightly lower (0.8401 and 0.9670), suggesting a moderate‐to‐strong fit. The lack‐of‐fit *p* values for UAE responses were also nonsignificant, indicating that the experimental data aligned well with the predictive models. Only the significant independent variables were used to derive model equations represented for MP‐UAE (1–4), MAE (5–8), and UAE (9–12).

Overall, MP‐UAE showed the highest model performance and prediction accuracy, followed by UAE and then MAE, highlighting MP‐UAE’s superiority for extracting bioactive compounds with consistent antioxidant activities from ER:

(1) MP‐UAETPC=182.4637.196.6219.264.644.3349.6721.8142.31−A−B+C+AC−BC−A2−B2−C2

(2) MP‐UAETFC=42.734.636.324.057.8312.483.693.62−A+B+C−AB−A2+B2−C2

(3) MP‐UAEDPPH=56.175.0210.274.47914.1610.934.925.8611.859.43−A−B+C−AB+AC−BC−A2+B2+C2

(4) MP‐UAEABTS=86.079.204.115.864.292.843.696.73−A−B+C−AB+AC+BC−A2

(5) MAETPC=172.5547.5115.0422.3417.514.9470.91+A+B+C+AC−BC−C2

(6) MAETFC=45.272.072.888.031.723.9410.93+A+B−AB−AC−BC−C2

(7) MAEDPPH=62.121.032.561.580.971.272.23+A+B−AB−BC+A2−B2

(8) MAEABTS=60.142.143.901.97+A+B+C

(9) UAETPC=77.6740.0426.385.9225.2532.0810.1756.2234.03+A+B+C−AC−BC+A2+B2+C2

(10) UAETFC=47.624.773.404.093.828.804.94+B+C+AB+BC+A2−C2

(11) UAEDPPH=59.067.664.034.986.374.21+A+B+BC+A2−B2

(12) UAEABTS=67.983.739.965.155.6710.665.30+B+AB−AC+A2−B2−C2

### 3.6. Optimization and Validation of the Model

A numerical optimization process was conducted using Design‐Expert software to optimize MAE and UAE. This process identifies a set of optimal conditions that maximize all responses collectively [64].

For all three techniques, all input factors were maintained “in range,” and among the solutions generated by the software, the one with the highest desirability factor was chosen as 1.00. The optimal conditions for MP‐UAE were determined to be 48 min of sonication time, 238 W power, and SPR of 19 mL/g, while for MAE, the optimal conditions were determined to be 311 W power and SPR of 30 mL/g and 51 s of microwave irradiation time. For UAE, the optimal settings were 60 min of extraction time, a 19 mL/g SPR, and a 36°C reaction temperature. Model accuracies were confirmed through validation experiments under these optimal conditions, with Table 3 presenting the predicted and observed responses. The MP‐UAE method demonstrated superior extraction efficiency compared to MAE and UAE, yielding higher TPC, TFC, and antioxidant activity. The synergy of these combined techniques also makes the system more economical, as it operates with lower microwave power, reduced extraction volume, and shorter sonication times than the individual methods.

### 3.7. Optimized Embelin Extraction

Embelin, a natural benzoquinone derivative mainly derived from the plant ER, has demonstrated significant antiviral properties [65]. Research suggests that embelin disrupts viral replication and inhibits enzymes crucial for viral life cycles [66]. Its antiviral actions include blocking viral entry, replication, and gene expression, making it a promising candidate for potential antiviral therapies [67, 68]. Table 9 compares three extraction techniques for embelin content (mg/g dry weight, DW), with MP‐UAE achieving the highest yield (39.98 ± 0.97 mg/g DW) followed by MAE and then UAE. This may be because it may have a greater ability to disrupt the cell structure, thereby enhancing mass transfer and significantly improving extraction efficiency.

**TABLE 9:** Estimation of embelin content in optimized extract (conditions reported in Table 3).

Techniques	Embelin content (mg/g DW)
MP‐UAE	39.98 ± 0.97
MAE	31.74 ± 0.32
UAE	27.96 ± 0.92

### 3.8. Molecular Docking of NS3/4A Protease With ER‐Selected Phytochemicals

The HCV NS3/4A protease is a key enzyme in HCV replication and immune evasion. It processes the viral polyprotein for replication and disrupts host immune responses by inhibiting interferon pathways. By cleaving proteins like MAVS and TRIF, it weakens the immune defense, aiding chronic infection. As a result, NS3/4A is a primary target for antiviral therapies against HCV [69, 70]. The initial evaluation of the docked complexes for the selected phytochemicals showed that, among the 11 compounds and boceprevir as the reference drug, four compounds were optimally positioned within the hotspot without any overlap or deformation. Amentoflavone, embelin, quercetin, and aloe‐emodin exhibited favorable interactions within the binding pocket (Figure 8). The root‐mean‐square deviation (RMSD) between the co‐crystal structure and the docked conformation is 0.7538 Å. This low RMSD indicates a close alignment, suggesting that the docking procedure accurately reproduced the experimental binding position [71].

**FIGURE 8:**
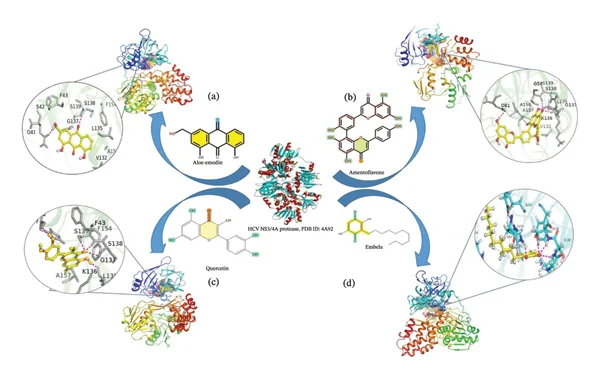
3D structures and interactions of docked compounds (Top‐4 compounds); (a) aloe‐emodin, (b) amentoflavone, (c) quercetin, (d) embelin.

Figure 8 shows 3D interactions of the docked compounds with NS3/4A protease (PDB 4A92). As shown in Table 4, amentoflavone, which exhibited the highest inhibition, achieved the top docking score of −8.991 kcal/mol among the docked configurations. Visual assessment of the docked complex of amentoflavone displayed noncovalent interactions (conventional hydrogen bonds) with amino acids PHE^486^, GLN^41^, GLY^137^, SER^138^, SER^139^, LEU^135^, ALA^157^, ASP^81^, and lipophilic interactions with amino acids HIS^57^ and MET^485^. The presence of hydrogen bonds in the amentoflavone–protease complex with critical amino acids, such as PHE^486^, GLN^41^, and SER^138^, underscores its specificity and stability, contributing to the overall binding strength and enhancing the compound’s potential efficacy as an inhibitor [72]. Furthermore, the lipophilic interactions with residues such as HIS57 and MET485 suggest that amentoflavone can achieve additional stability through hydrophobic contacts, which are essential for ensuring a robust interaction within the binding pocket [73]. These interactions not only highlight the significance of amentoflavone in inhibiting the HCV NS3/4A protease but also point to potential strategies for optimizing this compound for therapeutic purposes. By identifying the key interacting residues, researchers can make informed modifications to improve binding affinity and specificity, potentially resulting in more effective bioactive/antiviral agents [22]. These interactions play an important role in the contact of the amentoflavone with the HCV NS3/4A serine protease [74].

Aloe‐emodin exhibited a docking score of −8.014 kcal/mol among the docked configurations. Illustrative inspection of the docked complex confirmed conventional hydrogen bonding interactions with amino acids GLN^41^, SER^138^, SER^139^, T‐shaped interaction with HIS^57^ and π‐alkyl interaction with ALA^157^ and LYS^136^. The formation of conventional hydrogen bonds with amino acids enhances the stability and specificity of the complex, and the T‐shaped interaction with HIS^57^ suggests favorable π‐π stacking, further anchoring the ligand within the binding pocket. Furthermore, the π–alkyl interactions with ALA^157^ and LYS^136^ highlight the importance of hydrophobic contacts in stabilizing the ligand–receptor complex [75].

Evaluation of the interactions of embelin and quercetin (docking score of −8.152 and −8.107 kcal/mol, respectively) with HCV NS3 protease binding pocket depicted different types of interactions. In the case of embelin, hydrogen bonding interactions are observed with amino acids GLY^137^, SER^138^, SER^139^, and ALA^157^ and the interaction with HIS^57^ may involve T‐shaped π–π stacking, highlighting the importance of this residue in effectively anchoring the ligand within the binding pocket. Additionally, the presence of hydrophobic residues such as ALA^156^ and ALA^157^ suggests π–alkyl interactions, which enhance binding through nonpolar contacts. The diagram also depicts an attractive charge interaction with VAL^524^, indicating that electrostatic interactions may further stabilize the complex. Furthermore, quercetin showed conventional hydrogen bonds, shown in red (Figure 9), established between the ligand and critical amino acids, including HIS^57^, SER^138^, SER^139^, GLY^137^, and GLN^41^. These hydrogen bonds play a crucial role in stabilizing the ligand within the binding pocket, thereby enhancing the specificity and strength of the ligand–protease complex. Additionally, the presence of hydrophobic residues like LEU^135^ and ALA^157^ suggests π–alkyl interactions, which strengthen the binding through nonpolar contacts.

**FIGURE 9:**
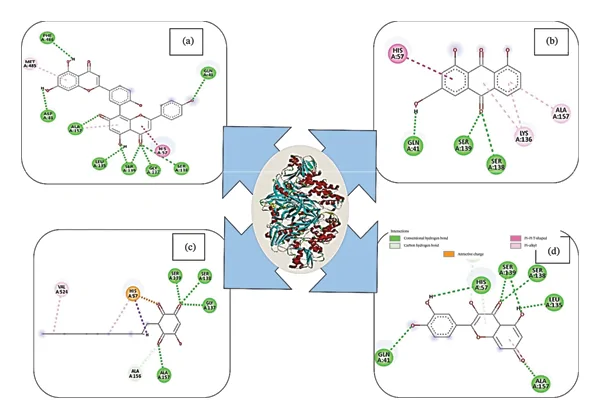
2D docking diagram (a) ER‐8, (b) ER‐9, (c) ER‐10, (d) ER‐4.

Collectively, these varied interactions emphasize the complexity and specificity of the ligand’s binding to the NS3/4A HCV protease, underscoring its potential as an effective inhibitor in the development of antiviral therapies.

#### 3.8.1. Pharmacokinetics and Toxicity Analysis

Table 5 outlines the toxicity parameters of selected compounds using the StopTox model, focusing on several toxicity indicators, including acute inhalation, oral, and dermal toxicity, as well as eye irritation, skin sensitization, and skin irritation. Most compounds, such as ER‐1, ER‐2, and ER‐5, are nontoxic in terms of acute inhalation, oral, and dermal toxicity, but show varying degrees of eye irritation and skin sensitization. Notably, ER‐3, ER‐7, ER‐11, and ER‐12 display acute oral toxicity, while ER‐4, ER‐6, and ER‐8 show acute dermal toxicity. Eye irritation and corrosion are prevalent in many compounds, such as ER‐1, ER‐2, and ER‐4. However, skin irritation and corrosion remain negative across all compounds. The varying sensitization properties indicate differing safety profiles, emphasizing the need for further analysis based on the compounds’ intended applications.

Figure 10 indicates BOILED‐egg model of selected ER‐phytochemicals showing a plot of molecular properties with WLOGP (hydrophobicity) on the *y*‐axis and TPSA on the *x*‐axis, commonly used to estimate drug‐likeness and membrane permeability [76]. The yellow ellipse represents molecules likely to cross the blood–brain barrier (BBB), while the white ellipse indicates human intestinal absorption (HIA). Molecules marked as PGP+ (blue circles) are substrates of P‐glycoprotein, affecting drug distribution, while PGP (red circles) are not. Molecules 3, 7, 12, and 11 fall within the BBB region, suggesting they may cross the barrier and are PGP + substrates, whereas molecules like 5, 10, and 13, with higher lipophilicity, are less likely to cross the BBB. Molecule 8 shows high hydrophobicity but high TPSA, reducing its membrane permeability, while Molecules 2 and 14, with high TPSA, are more polar and less likely to permeate biological membranes. The plot helps visualize the permeability potential and P‐glycoprotein interactions of different molecules.

**FIGURE 10:**
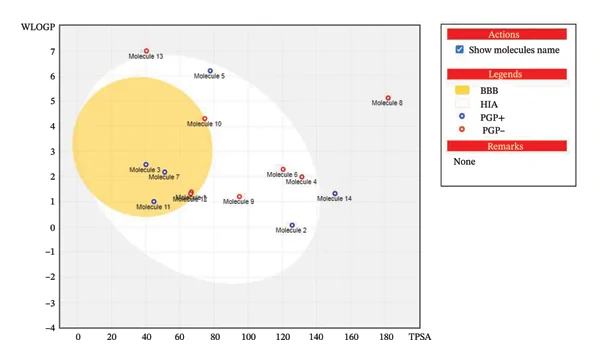
BOILED‐egg model of ER‐phytochemical.

Molecules with the highest docking score (ER‐8, ER‐9, ER‐10, and ER‐4) show varying properties relevant to their potential as HCV NS3 protease inhibitors. ER‐8, with high hydrophobicity (WLOGP) and moderate polar surface area (TPSA), may interact well with hydrophobic regions of the protease, but its higher TPSA could limit membrane permeability and bioavailability. ER‐9, being less hydrophobic and with moderate TPSA, appears better suited for intestinal absorption, making it a promising candidate for oral administration, with a balanced profile for solubility and protease interaction. ER‐10, more lipophilic than ER‐9, might have stronger interactions with the protease but could face solubility issues despite being in the HIA region, indicating good absorption potential. Molecule 4, with lower WLOGP and moderate TPSA, strikes a favorable balance between hydrophilicity and lipophilicity, suggesting good absorption and permeability, making it a strong candidate for effective NS3 protease inhibition. Overall, Molecules 9 and 4 seem to offer the best balance for bioavailability and inhibition potential, while Molecules 8 and 10 may require optimization for solubility and permeability.

## 4. Conclusion

In conclusion, this study demonstrates the superior efficiency of MP‐UAE as a green and optimized technique for recovering bioactive compounds from ER seeds. Under optimized conditions, MP‐UAE yielded the highest TPC (183.00 mg GAE/g DW), TFC (54.54 mg RE/g DW), DRSA (72.80%), and ARSA (81.33%), clearly outperforming UAE and MAE. Notably, embelin yield was also maximized (39.98 ± 0.97 mg/g DW), confirming the effectiveness of this hybrid extraction approach. Molecular docking results further revealed that key phytochemicals, including amentoflavone (−8.991 kcal/mol), embelin (−8.152 kcal/mol), quercetin (−8.107 kcal/mol), and aloe‐emodin (−8.014 kcal/mol), exhibited stronger binding affinities toward HCV NS3 serine protease than the reference drug boceprevir (−5.342 kcal/mol), underscoring their promising antiviral potential. The novelty of this work lies in the integration of an advanced green extraction technique with in silico antiviral screening, providing a dual approach to enhance both yield and functional relevance of phytochemicals. These findings not only validate MP‐UAE as an efficient extraction strategy but also position *E. ribes*–derived compounds as potential candidates for antiviral drug development. Future research should focus on in vitro and in vivo validation of antiviral activity, detailed pharmacokinetic and toxicity profiling of embelin and other lead compounds, and scale‐up studies to evaluate industrial feasibility. Additionally, exploring synergistic interactions among phytochemicals and expanding computational studies to other viral targets could further strengthen the therapeutic potential of this plant.

## Funding

This study was supported by the Princess Nourah bint Abdulrahman University Researchers Supporting Project number (PNURSP2026R158), Princess Nourah bint Abdulrahman University, Riyadh, Saudi Arabia.

## Disclosure

All authors read and approved the final manuscript.

## Ethics Statement

This study did not involve the use of human participants or animal subjects.

## Conflicts of Interest

The authors declare no conflicts of interest.

## Data Availability

The data supporting the findings of this study are available from the corresponding author upon reasonable request.
